# Managing Crohn’s Disease Postoperative Recurrence Beyond Prophylaxis: A Comprehensive Review with Meta-Analysis

**DOI:** 10.3390/biomedicines12112434

**Published:** 2024-10-23

**Authors:** Andrei Ovidiu Olteanu, Artsiom Klimko, Eugen Nicolae Tieranu, Andreea Daniela Bota, Carmen Monica Preda, Ioana Tieranu, Christopher Pavel, Mihai Radu Pahomeanu, Cristian Valentin Toma, Adrian Saftoiu, Elena Mirela Ionescu, Cristian George Tieranu

**Affiliations:** 1 Department of Gastroenterology, “Carol Davila” University of Medicine and Pharmacy, 020021 Bucharest, Romania; ovidiu-andrei.olteanu@drd.umfcd.ro (A.O.O.); carmen.preda@umfcd.ro (C.M.P.); christopher.pavel@gmail.com (C.P.); adrian.saftoiu@umfcd.ro (A.S.); mirela.ionescu@umfcd.ro (E.M.I.); cristian.tieranu@umfcd.ro (C.G.T.); 2 Department of Gastroenterology, ELIAS Emergency University Hospital, 011461 Bucharest, Romania; 3 Laboratory of Molecular Neuro-Oncology, Department of Neurology, University Hospital Zurich, 8091 Zurich, Switzerland; 4 Department of Cardiology, University of Medicine and Pharmacy, 200349 Craiova, Romania; 5 Department of Gastroenterology, Fundeni Clinical Institute, 022328 Bucharest, Romania; 6 Department of Pediatrics, “Marie Sklodowska Curie” Children Emergency Hospital, 077120 Bucharest, Romania; 7 Department of Gastroenterology, Clinical Emergency University Hospital, 014461 Bucharest, Romania; 8 Department of Internal Medicine and Gastroenterology, Bucharest Emergency University Hospital, 050098 Bucharest, Romania; 9 Department of Innovation and e-Health, “Carol Davila” University of Medicine and Pharmacy, 020021 Bucharest, Romania; cristian.toma@umfcd.ro

**Keywords:** postoperative recurrence, Crohn’s disease, biologic therapy, infliximab, ustekinumab, azathioprine, anti-TNF agents, endoscopic remission

## Abstract

Background and Aims: Postoperative recurrence in Crohn’s disease remains a significant clinical challenge, with high recurrence rates despite advancements in medical therapy. This study aims to evaluate the efficacy of various treatments for managing postoperative recurrence following ileocolonic resection in Crohn’s disease. Methods: A comprehensive search of PubMed, Cochrane, and Scopus databases was performed to identify studies reporting on the therapeutic management of postoperative recurrence in Crohn’s disease. Studies encompassing patients with an endoscopic Rutgeerts score of at least I2 were included. Results: Ustekinumab showed promise, achieving significant endoscopic and clinical success in difficult-to-treat patients. Anti-TNF agents demonstrated superior endoscopic and clinical remission rates compared to mesalamine and azathioprine. Retreatment with anti-TNF therapy remained effective even after preoperative failure. Thalidomide showed efficacy in refractory Crohn’s disease, but carries significant toxicity risks, necessitating careful patient selection and monitoring. Combination therapies and non-pharmacologic strategies like enteral nutrition offer additional options, though patient compliance remains challenging. Conclusions: Personalized treatment plans based on individual risk factors and biomarkers are crucial. Infliximab is recommended as the first-line treatment, with ustekinumab and vedolizumab as alternatives in case of anti-TNF failure or intolerance. Early intervention, patient education, and ongoing evaluation are essential for optimizing long-term outcomes in managing postoperative recurrence in Crohn’s disease.

## 1. Introduction

Crohn’s disease (CD) is a chronic, relapsing autoimmune inflammatory bowel disease that can affect any part of the gastrointestinal tract. It is phenotypically classified into inflammatory, stricturing, or penetrating types [1]. Despite significant advancements in medical therapy, approximately 75% of patients with penetrating or stricturing CD affecting the terminal ileum and/or right colon will require intestinal resection at some point [2]. Even after resection, recurrence is common [3]. The Third European Evidence-based Consensus on the Diagnosis and Management of Crohn’s Disease of the European Crohn’s and Colitis Organization (ECCO) defines recurrence as the reappearance of lesions after surgical resection, while relapse is a flare of symptoms in a patient previously in clinical remission [4].

Several risk factors for postoperative recurrence (POR) of CD are well established. These include younger age at diagnosis, active smoking, ileal disease, a perforating phenotype, and repeated surgeries due to CD [3]. Additional clinical risk factors are active perianal disease, continuous ileocolonic disease, and the NOD2/CARD15 genetic variant. Histological markers such as positive resection margins, granulomas in the resected tissue, and myenteric and submucosal plexitis also predict POR [5]. Bacterial dysbiosis and low levels of *Faecalibacterium prausnitzii* in both resected and postoperative ileal mucosa are associated with an increased risk of endoscopic recurrence, although antibiotic use in the perioperative period can affect the utility of microbiota in predicting recurrence [6].

The American Gastroenterological Association (AGA) defines high-risk patients for POR as those with ≥1 of the following: age at diagnosis < 30 years, current smoking, ≥2 previous surgeries for penetrating disease, with or without perianal disease. Low-risk patients are those diagnosed at >50 years of age, non-smokers, and with a first surgery for a short segment (10–20 cm) of fibrostenotic disease after a longer disease duration (>10 years) [7]. The ECCO guidelines are similar, defining high-risk patients for POR as those with ≥1 of the following: smoking, prior intestinal surgery, absence of prophylactic treatment, penetrating disease at index surgery, perianal location, granulomas in resection specimen, and myenteric plexitis [8].

After ileocecal resection, postoperative endoscopic recurrence without treatment occurs in approximately 65% to 90% of patients within one year and 80% to 100% within three years [9]. Histological recurrence can be detected in all patients within the first week post-surgery, with more than 70% developing endoscopic recurrence in the first postoperative year and up to 35% experiencing clinical relapse after three years. As postoperative endoscopic recurrence typically precedes clinical relapse, ileocolonoscopy is the gold standard for assessing POR of CD and is recommended 6–12 months post-surgery [3]. The Rutgeerts score (RS) evaluates the severity of endoscopic recurrence in postoperative CD patients and is a predictor of future clinical relapse [7,10]. The RS assesses the progression risk of CD based on the postsurgical endoscopic appearance, grading the severity of endoscopic lesions in the neo-terminal ileum and ileocolonic anastomosis. A score of ≥i1 signifies endoscopic recurrence, and escalation of medical therapy is recommended for scores ≥i2. A score of ≥i2 is a reliable predictor of subsequent clinical recurrence [7]. Thus early postoperative reduction of endoscopic inflammation with medications in this scenario can delay symptomatic recurrence [10]. Recently, lesions confined to the anastomosis have been separated from those in the neo-terminal ileum in a modified RS, with subscores i2a (anastomosis lesions) and i2b (>5 aphthous lesions in the neo-terminal ileum) [6]. The implementation of this modified score has yielded contradictory results, and until further prospective trials are available, the same treatment strategy should be applied to all i2 group patients [11].

ECCO and AGA guidelines are also concordant in recommending medical prophylaxis with thiopurines or anti-TNF drugs for patients with at least one risk factor for recurrence. Other options include high-dose mesalamine for patients with isolated ileal resection and imidazole antibiotics, which are less well tolerated. Addressing risk factors is crucial, with smoking cessation being particularly important [8,12]. In severe recurrence or complications, surgery may be necessary. The technique of anastomosis is also critical; Kono et al. introduced Kono-S anastomosis, which is based on the theory that inflammation in CD begins in the mesentery, and thus the anastomosis should be created away from it [13]. A randomized control trial comparing Kono-S anastomosis with conventional side-to-side anastomosis showed significant reductions in postoperative surgical and endoscopic recurrence rates and lower clinical recurrence rates, with no safety issues [14].

The management of POR in CD involves endoscopic monitoring, medical prophylaxis, and occasionally surgical intervention. The goals of treatment in CD are to achieve and maintain remission, improve long-term prognosis by limiting complications, and enhance the patient’s quality of life [10]. Traditionally, the focus was on inducing and maintaining symptomatic remission, but this approach did not prevent bowel damage or alter disease progression. Studies indicate that up to 50% of patients in clinical remission still exhibit objective inflammation [15]. Treatment goals have evolved with the STRIDE consensus to include ‘deep remission’—achieving both symptomatic and endoscopic remission [16].

Although numerous studies have examined the effects of prophylactic therapy on maintaining post-surgery remission, data on treating the established endoscopic POR to prevent clinical relapse are less consistent. This study aims to evaluate the efficacy and safety of different management strategies for POR of CD through a comprehensive review of the current literature.

## 2. Literature Review—Search Strategy

**Figure 1:**
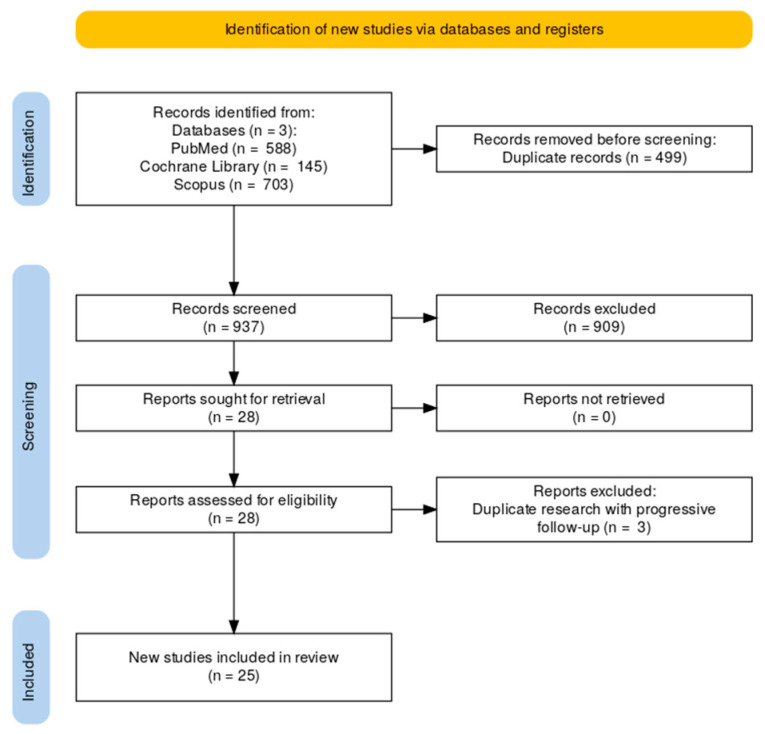
Flow chart showing the PRISMA methodology.

This review was conducted following the guidelines of the Preferred Reporting Items for Systematic Reviews and Meta-Analyses group (PRISMA) (Figure 1) in order to capture as many papers of interest as possible. A comprehensive search of PubMed, Cochrane, and Scopus databases was performed in March 2024. The search strategy included: (post-operative OR postoperative OR “post operative” OR “ileocolic resection” OR “ileo-colic resection” OR “ileocolonic resection” OR “ileo-colonic resection”) AND recurrence AND Crohn’s AND (biologic OR biologics OR “anti-tumor necrosis factor” OR “anti-tumour necrosis factor” OR anti-TNF OR infliximab OR adalimumab OR vedolizumab OR ustekinumab OR azathioprine OR 5-aminosalicylates).

We included studies that met the following eligibility criteria: (1) reporting data on the therapeutic management of established POR after ileocolonic resection in CD, (2) defining POR as RS ≥i2, and (3) study types including case reports, retrospective/prospective studies, and randomized controlled trials (RCTs). Excluded studies were those referring strictly to prophylaxis of POR and reviews.

Two authors independently reviewed all titles and abstracts to identify relevant studies, selecting articles that met the inclusion and exclusion criteria. They used Rayyan software, available online at https://new.rayyan.ai. Duplicates were semi-automatically removed by the software, after double-checking with the reviewers. For discrepant decisions between the two individual researchers, a third researcher reviewed the full-text papers and decided upon inclusion.

## 3. Meta-Analysis Methodology and Risk of Bias Assessment

We decided to perform a network meta-analysis (NMA) to compare the efficacy of different treatments for postoperative recurrence (POR) of Crohn’s disease. The analysis was performed using R software within the RStudio integrated development environment (Version 2024.9.0.375), specifically the “netmeta” package version 2.9.0. [17]. The included studies met the following criteria: (a) reporting on therapeutic management of postoperative recurrence in Crohn’s disease following ileocolonic resection and (b)reporting the endoscopic Rutgeerts score of at least i2.

The primary outcome selected was endoscopic improvement, defined as a reduction of at least one point in the Rutgeerts score. For the studied outcome, the odds ratios (ORs) with 95% confidence intervals (CIs) were calculated using a random-effects model to account for heterogeneity across studies.

**Figure 2:**
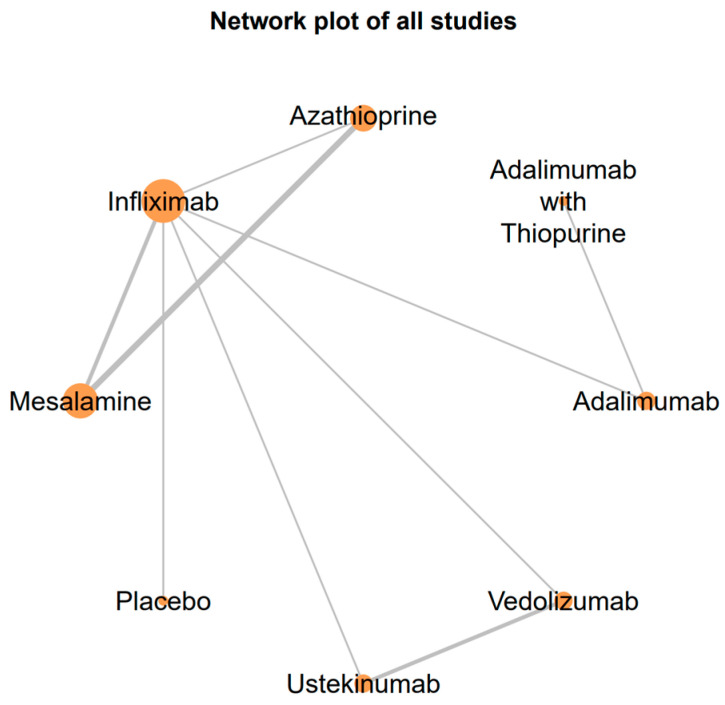
The network plot of included treatments illustrates the comparative relationships between treatments analyzed in the network meta-analysis. Each node represents a treatment, with the size of the nodes corresponding to the number of studies that examined each treatment. The edges (lines) connecting the nodes represent direct comparisons made between treatments, with the thickness of the edges indicating the number of studies that made each comparison. IFX is centrally located, which highlights its extensive evaluation against various other treatments.

**Figure 3:**
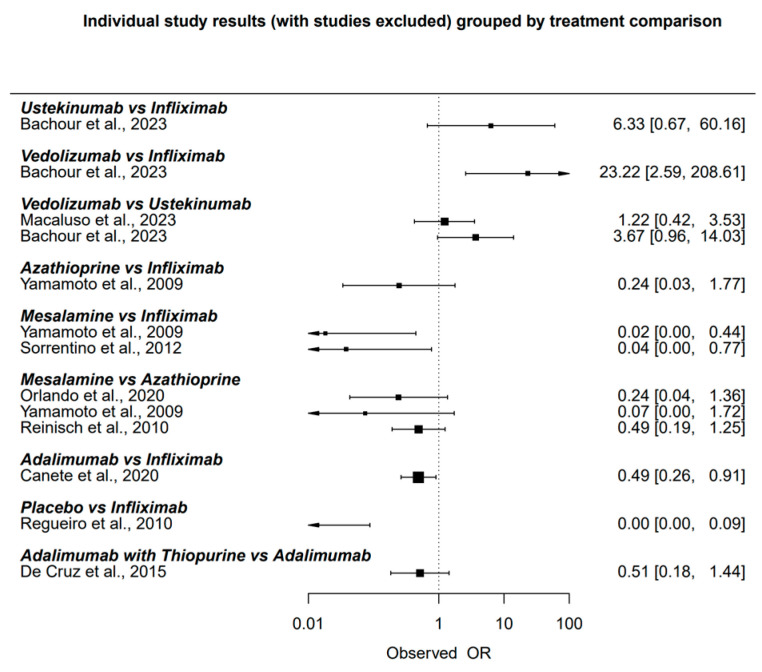
Individual study results grouped by treatment comparison [10,18,19,20,21,22,23,24,25].

The “netmeta” package in RStudio was used to create a network plot, illustrating the direct and indirect comparisons between treatments. The relative treatment effects were estimated using a frequentist approach, which calculates odds ratios for each treatment comparison. The network plot (Figure 2) and the forest plot (Figure 3) were generated using the same software package to visually present the relationships between treatments and the comparative odds ratios.

To verify the validity of our findings, we performed a risk of bias assessment. The quality of the included randomized controlled trials (RCTs) was assessed using the Cochrane Risk of Bias tool, while cohort and case-control studies were evaluated using the Newcastle-Ottawa Scale. Case reports were assigned, by default, a high risk of bias due to various reasons such as lack of control group, selection bias, confounding factors, and lack of blinding. Studies with a high risk of bias or insufficient reporting of endoscopic outcomes were excluded from the meta-analysis [26,27].

## 4. Results

A total of 1436 studies were initially identified (588 Pubmed, 145 Cochrane, and 703 Scopus). After the elimination of duplicates, 937 studies were assessed for eligibility, and 25 were included in the final review. The primary reason for such a high exclusion rate (909 articles, 97.01%) was the specific focus of these papers limited to prophylactic measures rather than the treatment of established POR.

The detailed study inclusion process can be summarized in the following flow diagram based on the PRISMA methodology (Figure 1).

The results are presented in Table 1, which includes the following columns: “Author and Study Year”, “Outcome”, “Type of Trial”, “Drug vs. Placebo/Drug Efficacy Comparison”, “Number of Patients”, “Endoscopic Response”, “Clinical Response”, “Primary Endpoint”, “Secondary Endpoint”, “Duration of Follow-up”, “Adverse Events”, and “Comments”. The primary endpoint was defined as the main outcome measure of each study, while the secondary endpoint included any additional outcomes used to evaluate the results. The “Comments” column in the review table provided additional context and insights that were not captured by the other columns, such as specific observations made by the authors, notable limitations, or unique findings relevant to the interpretation of the study results.

This search strategy ultimately identified 25 eligible studies, encompassing randomized controlled trials (RCTs), cohort studies, and case reports, focusing on the therapeutic management of established POR of CD after ileocolonic resection. The total number of patients across these studies was 2140, with a diverse range of follow-up durations and endpoints. The average follow-up duration varied significantly, with some studies providing short-term (6 months) and others long-term (up to 10 years) outcomes. The common endpoints evaluated included endoscopic improvement, clinical remission, and the incidence of adverse events.

The studies included multiple designs: 6 randomized controlled trials (RCTs), 5 retrospective cohort studies, 2 prospective cohort studies, 2 case reports, 1 case-control study, and 9 other various study types including observational studies, pilot studies, and follow-up surveys. These studies collectively involved patients with moderate to severe CD, primarily focusing on those with RS ≥ i2, consistent with the diagnosis of endoscopic POR. Patient demographics were diverse, with many studies including patients who had undergone previous resections and those with a history of failure of prior biological therapies.

The primary outcomes measured were endoscopic and clinical responses. Endoscopic improvement was typically defined as a reduction in the RS with at least 1 point, while clinical remission was measured by the absence of clinical symptoms and the need for additional intervention. For example, Macaluso et al. (2023) reported that 50% of patients achieved a reduction of at least one point in the RS, and 72.7% attained clinical success with UST [18]. Similarly, Bachour et al. (2023) noted that 60% of patients treated with UST achieved endoscopic improvement, and 55% achieved clinical remission at 12 months [19].

The efficacy of different treatments varied across the studies. Azathioprine (AZA) and high-dose 5-aminosalicylic acid (5-ASA) were compared in several studies, with AZA generally showing higher rates of endoscopic and clinical improvement but also a higher incidence of adverse events [20,24]. Anti-TNF agents, such as infliximab (IFX) and adalimumab (ADA), demonstrated superior efficacy compared to mesalamine and AZA in multiple studies, with higher rates of endoscopic remission and clinical success [10,23].

Biologic therapies, including ustekinumab (UST), vedolizumab (VDZ), and anti-TNF agents, showed significant promise in managing POR. UST was particularly effective, with studies showing significant rates of endoscopic and clinical success [18,19]. VDZ also showed potential effectiveness, with 47.6% of patients achieving endoscopic success and a clinical failure rate of 19.0% at one year [32].

Combination therapies, particularly those involving anti-TNF agents and immunomodulators, were found to be more effective than monotherapy. Huinink et al. (2023) reported that combination therapy significantly reduced the treatment failure rate at 2 years compared to anti-TNF monotherapy (30% vs. 49%, *p* = 0.02) [28]. This highlights the importance of combination therapy in maintaining remission and reducing the need for additional interventions.

Non-pharmacologic strategies, such as enteral nutrition (EN), also played a role in managing POR. Yamamoto et al. (2012) demonstrated that EN significantly reduced the incidence of recurrence requiring biologic therapy or reoperation compared to the control, though compliance issues were noted [37]. Novel treatments like thalidomide and ruxolitinib were explored for their potential in refractory cases. Thalidomide was effective in inducing mucosal healing in a case report by Hu et al. (2016), and ruxolitinib showed significant clinical and endoscopic improvement in a single patient [31,34].

Adverse events varied across the studies, with some treatments associated with higher rates of adverse reactions. For example, AZA was linked to myelosuppression, hepatotoxicity, and increased risk of lymphoma, while thalidomide posed risks of teratogenicity and peripheral neuropathy [24,43]. However, some biologics like UST reported no serious adverse events, underscoring their favorable safety profile [18].

We conducted a network meta-analysis enrolling nine studies with a total of 561 patients to determine which clinical treatment is most likely to produce an endoscopic response, defined as an improvement of at least one point in the RS. The studies included in our initial study were highly heterogeneous, with varied definitions of clinical response, relapse, and therapeutic failure. Therefore, we focused on more objective outcomes like the endoscopic response, as the definitions were more consistent across studies, which limited the number of studies included.

For this shortlist analysis, we excluded case reports, incomplete abstracts from congress presentations, and studies with variable step-up treatment protocols, where patients were initially treated with one drug and subsequently added another upon relapse. Additionally, studies that segregated patients into low- or high-risk cohorts with conditional treatment plans were excluded.

The network plot (Figure 2) demonstrates the interconnectedness of the various treatments evaluated. IFX was compared with multiple therapies, including AZA, mesalamine, ADA, UST, and VDZ. The plot underscores the comprehensive nature of the analysis and highlights the robust network of evidence supporting these findings.

Figure 3 provides an overview of the observed odds ratios (OR) and the comparative effectiveness of the treatments in inducing endoscopic response.

A frequentist network meta-analysis was performed, with IFX chosen as the reference treatment. IFX was selected as the reference drug because it is widely used and well studied, minimizing the effects of outliers and providing a robust baseline for comparison.

**Figure 4:**
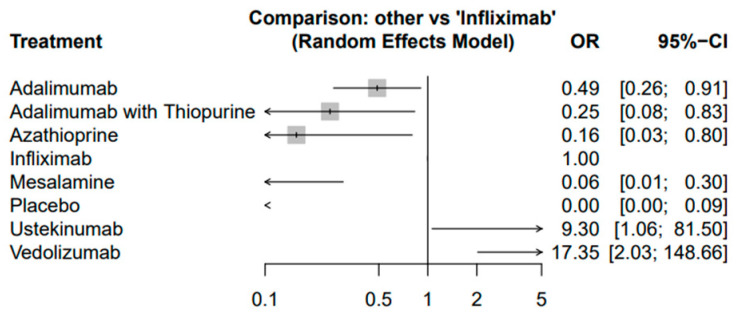
Random effects model—comparison of treatments to infliximab.

The network meta-analysis revealed that compared to IFX, several treatments showed varying degrees of effectiveness in producing endoscopic response (Figure 4). Adalimumab (OR: 0.49; 95% CI: 0.26–0.91), ADA combined with thiopurine (OR: 0.25; 95% CI: 0.08–0.83), and AZA (OR: 0.16; 95% CI: 0.03–0.80) were all associated with significantly lower odds of achieving endoscopic response compared to IFX. Conversely, UST (OR: 9.30; 95% CI: 1.06–81.50) and VDZ (OR: 17.35; 95% CI: 2.03–148.66) demonstrated higher odds of achieving endoscopic response, suggesting their potential efficacy in this setting.

However, this last observation should be interpreted with restraint due to the low number of patients in these studies. An overview of these results is presented in Table 2.

In the current review, the risk of bias assessment for the randomized controlled trials (RCTs) was performed using the Cochrane Risk of Bias 2 (RoB 2) tool, while observational studies were assessed using the Newcastle-Ottawa Scale (NOS). Overall, the majority of RCTs demonstrated a low to moderate risk of bias. The existing randomization processes reduced performance and selection bias in most trials. Possible drawbacks regarding missing outcome data were present in several studies, particularly in Regueiro et al. (2009) [25] and Cruz et al. (2014) [41], where long follow-up periods generated the potential for attrition bias. The outcomes, measured using standardized criteria like the Rutgeerts score, were similarly applied across studies, thus reducing measurement bias.

For observational studies assessed using NOS, the majority showed a low to moderate risk of bias. Well-defined cohorts and control for confounders were the main attributes mitigating the risk of bias. However, studies like Yamamoto et al. (2009) [10] and Sorrentino et al. (2012) [44] faced a moderate risk of bias due to the absence of control groups and limited adjustment for confounders.

Lastly, the two included case reports, Marques Cami et al. (2022) [31] and Hu et al. (2016) [34], while thorough in their documentation of patient information and outcomes, carry an inherently high risk of bias due to their descriptive nature, lack of control groups, and absence of blinding.

The overall low to moderate risk of bias in both randomized controlled trials and observational studies included in this review suggests that the findings provide a reliable foundation for clinical decision-making. Even though there were limitations identified, particularly in studies with moderate bias, the use of standardized outcome measures, such as the Rutgeerts score, reinforces the reliability of the results.

## 5. Discussion

POR in CD remains a significant clinical challenge, as surgery is not curative, and recurrence rates remain high despite advancements in medical therapy. Despite numerous studies exploring the efficacy of various treatments in preventing and managing POR, there is a lack of consensus on how to manage relapsing patients. The severity of endoscopic lesions within the first year following ileocolic resection is a well-documented predictor of clinical recurrence. Patients with endoscopic recurrence (ER) higher than i1 in the neoterminal ileum face an increased risk of early symptoms and complications. A significant proportion of ER is detectable as early as six months post-resection, with many cases being very severe [31].

### 5.1. 5-Aminosalicylates Versus Azathioprine

Once severe ER is documented, it is crucial to treat patients to avoid clinical and surgical recurrence. Studies have shown the efficacy of 5-ASA or AZA versus placebo in preventing clinical recurrence after surgically induced remission. However, only one previous randomized controlled trial compared the efficacy of 5-ASA versus AZA for clinical recurrence prevention in CD patients with severe postsurgical ER [20].

A multicenter randomized double-blind double-dummy trial by Orlando et al. (2020) evaluated the efficacy of high-dose 5-ASA and AZA in 46 patients with early severe postsurgical endoscopic recurrence (ER) (RS ≥ i2). In this high-risk population, 17.4% of patients experienced therapeutic failure, defined as clinical recurrence or drug discontinuation due to adverse events, within 12 months from randomization, with no significant difference between the 5-ASA and AZA groups (20.8% vs. 13.6%, respectively, *p* = 0.702). Therapeutic failure with 5-ASA was primarily due to clinical recurrence, while with AZA, it was related to adverse events. Additionally, AZA led to an RS decrease of ≥2 points in 27.3% and ≥1 point in 36.4% of patients, whereas 5-ASA achieved similar decreases in only 8.3% of patients. Clinical recurrence was encountered only in 5-ASA treated patients (20.8%), while AZA had a less favorable safety profile, with three adverse events leading to drug discontinuation, indicating a trade-off between efficacy and safety. Clinical relapse rates were 17% at 12 months and 53% up to 10 years post-trial, with no significant adverse events recorded [20]. A similar randomized double-blind double-dummy multicenter trial involving 78 patients found that AZA patients showed a ≥1 point reduction in the RS in 63.3% of cases, compared to 34.4% for mesalamine. Additionally, 5-ASA was less effective than AZA in preventing clinical recurrence (*p* = 0.031). Therapeutic failure was higher in the AZA group (22.0%) compared to the mesalamine group (10.8%), with adverse drug reactions leading to discontinuation in all these AZA patients [24]. AZA appears to have superior efficacy in reducing ER compared to mesalamine, although its use is limited by a higher incidence of adverse events.

The combination of mesalamine and thiopurine did not significantly enhance therapeutic outcomes compared to thiopurine alone. Endoscopic improvement was seen in 49% of patients, with no differences between the groups. Clinical recurrence occurred in 32% of cases and 11% of controls, with no specific adverse effects reported, indicating that adding mesalamine to thiopurine therapy does not significantly improve outcomes in POR management [20].

### 5.2. AntiTNF Agents Versus Azathioprine and 5-Aminosalicylates

The adverse event profile that limits the use of thiopurines, such as AZA, includes myelosuppression, hepatotoxicity, and increased risk of lymphoma, which often preclude adherence [34]. Within this context, an IFX-based regimen may be more effective than AZA/5-ASA protocols in reducing clinical and endoscopic disease activity. Yamamoto et al. found that six months after treatment, none of the IFX-treated patients developed postoperative clinical recurrence (PO-CR) compared with 38% of AZA-treated and 70% of 5-ASA-treated patients. Endoscopic inflammation improved in 75% of IFX-treated vs. 38% of AZA-treated and none of the 5-ASA-treated patients. Complete mucosal healing was achieved in 38% of IFX patients, 13% of AZA patients, and none of the 5-ASA patients. This reinforces the superiority of IFX in managing early endoscopic lesions and preventing clinical recurrence [10]. Treatment with mesalamine alone, although safer than AZA, is less effective—in a comparison study, 7 out of 13 patients treated with IFX achieved remission, while none did while being treated with mesalamine [23].

Moreover, retreatment with anti-TNF therapy, such as IFX or adalimumab (ADA), even after preoperative anti-TNF therapy failure, can be an effective strategy for managing postoperative CD. Adverse events were reported in 15% of patients receiving combination therapy and 18% receiving monotherapy, indicating a comparable safety profile. This finding is particularly important for patients who have previously failed anti-TNF therapy, suggesting that retreatment remains a viable option [28].

Over the past decade, the use of biological drugs, especially anti-TNFs, has increased for the prevention and treatment of postsurgical recurrence. This has led to the ECCO guidelines introducing the recommendation for prophylactic treatment with thiopurines or anti-TNFs after ileocolonic resection in patients with at least one risk factor for postsurgical recurrence. However, limited data are available on managing severe postsurgical ER. The POCER study found step-up treatment, meaning escalating therapy from ‘no treatment’ to a thiopurine or adalimumab, for early severe ER to be the best option for preventing clinical recurrence [22]. Compared to metronidazole alone, the active treatment approach improved endoscopic remission (33% vs. 51%) and recurrence (67% vs. 49%) profiles at 18 months. Within this trial, a higher proportion of patients in the 5-ASA group underwent treatment escalation compared to the AZA group, affirming the limited efficacy of mesalamine. Patients with severe postsurgical ER benefitted from prompt postsurgical treatment, as early AZA use did affect long-term clinical outcomes in high-risk patients [22].

Anti-TNF therapy may have some efficacy in preventing as well as in treating POR in CD. In a prospective, single-center, open-label pilot study, Papamichael et al. (2012) assessed ADA’s short- and long-term efficacy in the postoperative setting for patients at high risk for early POR and in patients with established POR. The study involved 23 patients treated with ADA following curative intestinal resection for complications of CD, divided into two subgroups of 8 and 15 patients respectively, receiving a standard induction regimen and maintenance therapy either straightaway following surgery or after endoscopic documentation of POR (RS ≥ 2). After one year of treatment, no clinical recurrences were observed, although 10% had PO-ER, and 45% had at least moderate histologic recurrence. Regarding the POR subgroup, at 24 months of ADA treatment, 60% of patients achieved complete or near-complete mucosal healing, and 56% maintained clinical remission [3].

Similarly, IFX in combination with low-dose oral methotrexate (10 mg/week) may have preventive potential for PO-ER and clinical recurrence of CD. None of the CD patients treated with scheduled IFX maintenance therapy in the immediate postoperative period experienced PO-ER for 24 months. However, 83% developed PO-ER within four months after discontinuing IFX. Re-treatment with lower doses of IFX successfully restored and maintained endoscopic remission for one year [45].

In the only randomized placebo-controlled trial to date, one-year treatment with the classical IFX regimen after surgery was far more effective than placebo in preventing endoscopic and histologic recurrence of CD (9.1% and 27.3% vs. 84.6% and 84.6%, respectively). The rate of PO-CR was likewise lower in the IFX arm compared to the placebo-treated patients (20.0% vs. 46.2%). This study also suggested that improved outcomes could be maintained in subjects who continued IFX therapy. Local injections of low-dose IFX into the sites of endoscopic recurrence prevented short- and medium-term clinical relapses of CD without compromising safety, improving both Rutgeerts endoscopic and histologic scores [25,38].

IFX was also more effective than AZA and mesalamine in managing early postoperative recurrence, as quantified by endoscopic improvement (75% with IFX, 38% with AZA, and 0% with mesalamine) and clinical recurrence (0% with IFX, 38% with AZA, and 70% with mesalamine) [10].

The comparative efficacy of IFX is higher than ADA in the postoperative period, which can be further augmented by thiopurines, resulting in higher trough levels of IFX and decreased underexposure rates. Canete et al. found that roughly 25 months after surgery, IFX patients had higher rates of endoscopic remission (57% vs. 29%) and marginally improved rates of clinical response (61% vs. 57%) compared to ADA patients [21].

Several studies further examined whether combinatorial anti-TNF therapy may augment outcomes. Postsurgical anti-TNF therapy reduced treatment failure rates by 19% at 2 years when combined with immunomodulators compared to anti-TNF monotherapy (30% vs. 49%, *p* = 0.02). The cumulative rates of treatment failure were 28% at 1 year and 47% at 2 years, with combination therapy proving superior in maintaining remission [28]. In an RCT, De Cruz et al. compared thiopurine/ADA versus placebo in 85 patients, finding that step-up treatment results in a 38% remission rate at 12 months., suggesting that the combination of thiopurine and ADA can provide significant benefits in early intervention, potentially reducing long-term recurrence rates [30].

### 5.3. New Biologic Agents

Ustekinumab (UST) shows promise as a therapeutic option for treating POR. In a multicenter real-world study, 50% of patients achieved endoscopic success, and 72.7% attained clinical success among difficult-to-treat patients, even those with high rates of previous resections and failures to prior biological therapies. Approximately 50% of patients achieved at least a one-point reduction in the RS, and 27.3% reported an absence of POR (RS ≤ i1) at the first post-treatment colonoscopy. UST may slow or halt the progression of POR, particularly for severe (i3 or i4) and intermediate severity (i2) lesions [18]. Similar results were reported by Bachour et al., where UST therapy for POR-CD resulted in a 60% endoscopic response and a 55% clinical remission rate at 12 months [19]. Both studies did not report any adverse events and present UST as a viable treatment option for patients with moderate to severe postsurgical disease activity [18,19].

When comparing UST to vedolizumab (VDZ), both drugs showed similar rates of endoscopic success (50.0% for UST vs. 47.6% for VDZ) and clinical failure (27.3% for UST vs. 32.8% for VDZ). However, UST demonstrated a slightly lower rate of surgical recurrence (9.1%) over a mean follow-up of approximately 18 months. UST may offer a slight advantage in terms of long-term outcomes, although further research is needed to confirm these findings and determine factors that could guide treatment selection [18]. VDZ monotherapy was assessed by Macaluso et al. in a 58-patient cohort study, where endoscopic success was 47.6% and clinical failure was 19.0% at one year, which increased to 32.8% at roughly two years [32].

### 5.4. Other Non-Biologic Agents

Several other treatment strategies have been explored for their efficacy in managing POR, including thalidomide and ruxolitinib. The ECCO/ESPGHAN consensus guidelines recommend thalidomide as an alternative for patients who do not tolerate or lose response to anti-TNF agents [46]. Simon et al. reported 54% of their 77 patients with active refractory CD to achieve clinical remission within the first year of thalidomide treatment [47]. Although thalidomide’s use in surgical CD is less documented, Hershfield et al. reported significant improvement in terminal ileal ulcers in a CD patient who failed other treatments [48]. There are also several case reports that document excellent responses to thalidomide in patients with refractory disease, with clinical remission being maintained for up to 15 months without adverse effects [34]. Thalidomide, similarly to thiopurines, is limited by its toxicity profile—the former drug may cause peripheral neuropathy, thromboembolism, sedation, and dermatitis. It is also a notorious teratogen [34].

Evidence examining ruxolitinib, a JAK inhibitor, is limited—a case report documented a 50% reduction in mucosal ulceration, along with a six-month clinical remission in a refractory POR patient [31].

Long-term enteral nutrition (EN) with an elemental diet showed promise in reducing recurrence incidence and the need for biologic therapy, although patient compliance remains a challenge. Motivated patients who have maintained EN therapy for over five years demonstrated its potential feasibility for long-term management in select populations. Yamamoto et al. (2012) demonstrated that EN significantly reduced the incidence of recurrence requiring biologic therapy or reoperation compared to controls in a cohort of 40 patients. Endoscopic recurrence was lower in the EN group (56%) compared to the control group (82%), and clinical recurrence was also reduced (30% vs. 60%). However, compliance issues were noted, impacting the long-term efficacy of EN [37].

Within this complex treatment landscape, identifying predictive factors and understanding long-term outcomes are critical for improving management strategies for POR. Treatment timing recommendations are somewhat discordant. The POCER study suggested that initiating optimum drug therapy based on endoscopic detection is preferable to waiting for clinical symptoms to appear [22]. Conversely, Riviere et al. found that using immunosuppressants and tumor necrosis factor antagonists to treat asymptomatic endoscopic postoperative recurrence of CD did not significantly reduce long-term clinical recurrence risk in patients with Rutgeerts scores of i2 but had a small effect in those with scores of i3 or i4. An RS ≥ i2 increases the risk of clinical and endoscopic CD recurrence. Riviere et al. reported clinical and surgical POR rates of 48% and 26%, respectively, within a median follow-up of 88 months [33].

The findings of our review suggest that IFX is the preferred treatment for managing early endoscopic lesions post-resection in CD patients, particularly those at high risk for severe endoscopic recurrence. Proactive monitoring and timely step-up therapeutic adjustments are integral to therapeutic success. AZA can be considered for patients at lower risk for adverse events and those preferring an immunomodulatory approach, while mesalamine should be limited to those with mild disease or contraindications to other treatments. EN therapy is a viable option for motivated patients who can maintain the regimen, with education and support enhancing compliance. For clinical practice, IFX is recommended as the first-line treatment for high-risk patients, with new biologic agents such as UST and VDZ as a second-line option in case of anti-TNF contraindications, failure, or intolerance. Moderate-risk patients may start with AZA, considering their risk profile and potential for adverse events, and mesalamine or long-term EN as alternatives for those with low risk and good compliance. Patients preferring non-pharmacologic approaches can consider long-term EN with an elemental diet, supported by educational and compliance strategies.

Our study has limitations that may interfere with the general applicability of our findings. Firstly, there is significant heterogeneity in defining clinical outcomes across studies. To address this, we focused our network meta-analysis on studies with endoscopic outcomes based on Rutgeerts scores (RS). Secondly, the low number of patients in studies evaluating the efficacy of vedolizumab (VDZ) and ustekinumab (UST) may falsely infer their superiority over infliximab (IFX). Lastly, variable follow-up intervals can influence drug efficacy interpretations, as initial suboptimal endoscopic responses may improve with longer follow-up.

## 6. Conclusions

Managing POR in CD remains challenging despite advancements in medical therapy. This review highlights the efficacy of biologic therapies, particularly IFX, in reducing recurrence rates. Personalized treatment plans based on individual risk factors and biomarkers are crucial. For high-risk patients, IFX is recommended as the first-line treatment, with UST and VDZ as second-line options. Early intervention and proactive monitoring are essential for optimizing long-term outcomes in the postoperative setting of CD.

## Figures and Tables

**Table 1:** Summary of the studies reporting on the management of Crohn’s disease postoperative recurrence.

Author and Study Year	Outcome	Type of Trial	Drug vs. Placebo/Drug Efficacy Comparison	Number of Patients	Endoscopic Response	Clinical Response	Primary Endpoint	Secondary Endpoint	Duration of Follow-up	Adverse Events	Comments
Macaluso et al., 2023 [18]	Endoscopic and clinical response	Real-world observational study	UST	44	50% achieved a reduction of at least one point in RS	72.7% clinical success	Reduction of at least one point in RS	Clinical success (absence of clinical failure)	17.8 ± 8.4 months	No adverse events reported	UST initiated for endoscopically documented POR, with significant rates of endoscopic and clinical success
Huinink et al., 2023 [28]	Retreatment with anti-TNF therapy for postoperative Crohn’s disease recurrence is valid. Combination therapy is more effective than monotherapy.	Retrospective cohort study	Anti-TNF therapy vs. combination therapy	364	Not specified	Not specified	Treatment failure rate (need for reintroduction of corticosteroids, immunosuppressants, or biologicals or need for re-resection)	Treatment failure rate at 1 and 2 years, analysis of preoperative anti-TNF failure, combination therapy vs. monotherapy, retreatment with the same or different anti-TNF agent	1 and 2 years	Not specified	Retreatment with anti-TNF therapy post-ICR is effective, especially with combination therapy. The study highlights the importance of combination therapy to reduce treatment failure rates
Bachour et al., 2023 [19]	Change in Biologic Class Promotes Endoscopic Remission Following Endoscopic Postoperative Crohn’s Disease Recurrence	Retrospective Cohort Study	New Biologic Class vs. Therapy Optimization/Continuation	81	Initiation of a new biologic class was associated with a higher rate of endoscopic improvement	60 patients (74.1%) experienced composite recurrence (persistent ePOR or surgical recurrence)	Composite endoscopic or surgical recurrence	Reduction of modified RS	Median follow-up from ePOR to subsequent endoscopy: 426.5 days	Not specified specifically for each intervention	The study emphasizes the benefit of changing the biologic class after the detection of ePOR despite prophylactic biologic therapy
Ueda et al., 2023 [29]	Endoscopic and clinical response	Retrospective cohort study	Biologic era treatments	267	Postoperative anastomotic lesions were detected in 61.0% at index ileocolonoscopy and 74.9% at follow-up ileocolonoscopy	Patients with intermediate or severe lesions required significantly more interventions (endoscopic dilation or surgery)	Frequency and severity of postoperative anastomotic lesions	Interventions required (endoscopic dilation or surgery)	~1 year, follow-up duration not specified	Not reported	Frequent and increasing severity of anastomotic lesions observed, prospective studies needed to evaluate treatment enhancement
De Cruz et al., 2022 [30]	Endoscopic and clinical response	Randomized controlled trial	Thiopurine/ADA vs. Placebo	85	A combination of ulcer depth and circumference at 6 months was associated with endoscopic recurrence at 18 months	38% remission at 12 months for patients who stepped up treatment at 6 months, 39% recurrence at 6 months	A combination of ulcer depth and circumference at 6 months was associated with endoscopic recurrence at 18 months	N/A	18 months	N/A	The combination of ulcer depth and circumference at anastomosis at 6 months was predictive of endoscopic recurrence at 18 months
Marques Cami et al., 2022 [31]	Endoscopic and clinical response	Case report	Ruxolitinib	1	More than 50% reduction of ulcerated mucosa in both ileocolonic anastomosis and neoileum	Clinical remission for six months, no further budesonide cycles needed	Reduction of ulcerated mucosa, clinical remission	Fecal calprotectin levels, blood test normalization	6 months	No adverse events reported	Patient showed significant clinical and endoscopic improvement related to ruxolitinib treatment, with satisfactory evolution of polycythemia vera
Macaluso et al., 2022 [32]	Endoscopic and clinical response	Cohort study	VDZ	58	Endoscopic success in 47.6% (reduction of at least one point of RS)	Clinical failure in 19.0% at one year, 32.8% at the end of follow-up, 12.1% required new resection	Endoscopic success (reduction of at least one point RS)	Clinical failure, need for new resection	Mean 24.8 ± 13.1 months	Not reported	VDZ shows potential effectiveness in treating POR of CD
Orlando et al., 2020 [20]	Endoscopic and clinical response	Randomized double-blind double-dummy trial	AZA vs. High-dose 5-aminosalicylic acid (5-ASA)	46	AZA: 6 (27.3%) with RS decrease ≥ 2 points, 8 (36.4%) with decrease ≥ 1 point; 5-ASA: 2 (8.3%) with RS decrease ≥ 2 points, 2 (8.3%) with decrease ≥ 1 point	AZA: 3 (13.6%) clinical recurrence; 5-ASA: 5 (20.8%) clinical recurrence	Therapeutic failure (clinical recurrence or drug discontinuation due to adverse events) at 12 months	10-year post-trial analysis of clinical and endoscopic outcomes	12 months	AZA: 3 adverse events leading to drug discontinuation (fever, hyperamylasemia, mild pancreatitis)	No significant difference in treatment failure between 5-ASA and AZA, AZA has a less favorable safety profile but may be more effective in preventing clinical recurrence
Canete et al., 2020 [21]	Endoscopic and clinical response	Multicenter retrospective observational study	IFX vs. ADA	179	Endoscopic improvement in 61%, endoscopic remission in 42%	59% clinical remission in patients with clinical POR at the start of therapy	Effectiveness of anti-TNF agents in improving mucosal lesions	Endoscopic improvement, clinical remission	Median 31 months (IQR 13–54)	Not specified	IFX showed higher rates of endoscopic response and remission compared to ADA; concomitant thiopurine use increased efficacy
Riviere et al., 2021 [33]	Clinical and surgical recurrence	Retrospective cohort study	Immunosuppressants and biologics	365	RS ≥ i2 associated with increased risk of clinical and surgical recurrence	48% clinical POR, 26% modified surgical POR within a median follow-up of 88 months	Clinical POR rates, surgical POR rates	Impact of endoscopy-guided therapy modification	Median 88 months	Not reported	RS ≥ i2 patients more likely to receive new therapy; modest decrease in clinical POR for RS i3 and i4 with immunosuppressants or biologics; no benefit for RS i2
Hu et al., 2016 [34]	Endoscopic and clinical response	Case report	Thalidomide	1	Mucosal healing achieved at 9 months; RS declined from i2 to i1	Clinical remission at 15 months	Mucosal healing (MH) of anastomotic ulcers	Endoscopic and clinical improvement	15 months	No adverse effects reported	Thalidomide is effective in inducing mucosal healing in postoperative CD endoscopic recurrence
De Cruz et al., 2015 [22]	Endoscopic and clinical response	Randomized controlled trial	Thiopurine/ADA vs. Metronidazole alone	174	60 (49%) in the active care group had endoscopic recurrence at 18 months vs. 35 (67%) in standard care	33 (27%) in the active care group had clinical recurrence (CDAI > 200) vs. 21 (40%) in standard care	Endoscopic recurrence at 18 months	Clinical recurrence, C-reactive protein levels, need for further surgery	18 months	No significant differences between active care and standard care groups	Early colonoscopy and treatment step-up for recurrence is better than conventional drug therapy alone
Zabana et al., 2014 [35]	Endoscopic and clinical response	Case-control study	Thiopurines with mesalamine vs. Thiopurines alone	37	Endoscopic improvement in 49%, no difference between groups	32% clinical recurrence in cases, 11% in controls (*p* = 0.2)	Development of clinical recurrence	Change in RS, mucosal lesions	Median 59 months (IQR 22–100)	No specific adverse effects reported for mesalamine	Mesalamine addition showed no benefit over thiopurine alone for endoscopic improvement or clinical recurrence rates
Reinisch et al., 2013 [36]	Clinical recurrence	Follow-up survey of randomized double-blind double-dummy trial	AZA vs. Mesalamine	46	N/A	36% clinical recurrence with AZA, 25% with mesalamine within 24 months post-treatment	Clinical recurrence within 24 months post-treatment	Long-term prevention of clinical recurrence	Approximately 4 years	N/A	No significant difference in time to clinical recurrence between AZA and mesalamine
Yamamoto et al., 2013 [37]	Endoscopic and clinical response	Prospective cohort study	Enteral nutrition (EN) vs. Control	40	56% (EN) vs. 82% (control) endoscopic recurrence	30% (EN) vs. 60% (control) clinical recurrence	Recurrence requiring biologic therapy or reoperation	Clinical recurrence rate, reoperation rate	5 years	Diarrhea and abdominal distension in the EN group	EN significantly reduced the incidence of recurrence requiring biologic therapy, though compliance issues noted
Papamichael et al., 2012 [3]	Endoscopic and clinical response	Prospective, single-center, open-label, two-year pilot study	ADA	23	60% (9/15) achieved complete (RS-i0) or near-complete (RS-i1) mucosal healing at 24 months	56% (5/9) of patients with clinical relapse at study enrolment achieved and maintained clinical and serological remission	Prevention of early (at 6 months) and late (at 24 months) PO-ER (Group I) and rate of complete mucosal healing (Group II)	Endoscopic and clinical improvement (Group II)	24 months	No serious adverse events reported	ADA is effective in preventing and treating PO-ER and PO-CR in high-risk CD patients
Sorrentino et al., 2012 [23]	Endoscopic and clinical response	Prospective open-label multicenter pilot study	IFX vs. Mesalamine	24	IFX: 54% endoscopic remission, 69% improvement in endoscopic score; Mesalamine: 0% endoscopic remission, no improvement in endoscopic score	IFX: 0% clinical recurrence; Mesalamine: 18% clinical recurrence at 8 and 9 months	Proportion of patients with endoscopic remission (score < 2) after 54 weeks	Improvements in endoscopic scores, clinical recurrence at 54 weeks	54 weeks	Flu-like symptoms in 3 patients in the IFX group, new positivity for anti-DNA and lupus anticoagulant antibodies in 2 patients	IFX is superior to mesalamine in treating postoperative endoscopic recurrence of CD, though prophylactic use of IFX may be more effective
Reinisch et al., 2010 [24]	Clinical and endoscopic recurrence	Randomized double-blind double-dummy multicenter trial	AZA vs. Mesalamine	78	63.3% of AZA patients showed ≥1 point reduction RS vs. 34.4% of mesalamine patients	22.0% therapeutic failure in the AZA group vs. 10.8% in the mesalamine group; clinical recurrence: 0% (AZA) vs. 10.8% (mesalamine)	Therapeutic failure during 1 year (CDAI ≥ 200 and increase of ≥60 points from baseline or drug discontinuation due to lack of efficacy/adverse reaction)	Endoscopic improvement at month 12, CDAI score change, IBDQ score change, CRP level change, mucosal healing	12 months	Adverse drug reactions led to discontinuation in 22.0% of AZA patients (e.g., pancreatitis, leucopenia)	AZA showed superior endoscopic improvement but higher adverse event-related discontinuations compared to mesalamine
Regueiro et al., 2010 [25]	Endoscopic and clinical response	Long-term follow-up of randomized controlled trial	IFX vs. Placebo	24	71% remission in the placebo group switched to INF at 2 years; recurrence in all INF patients who stopped at 1 year	Not specified	Long-term endoscopic remission and recurrence rates after surgery	Effectiveness of INF beyond the first postoperative year, response to INF after recurrence	Up to 4.5 years	Infusion reactions leading to switch to (ADA) in some patients	INF maintains remission with ongoing infusions; recurrence if stopped; effective in treating endoscopic recurrence in anti-TNF naive patients post-surgery
Yamamoto et al., 2009 [10]	Endoscopic and clinical response	Prospective pilot study	IFX vs. Mesalamine vs. AZA	26	75% endoscopic improvement with IFX, 38% with AZA, 0% with mesalamine	0% clinical recurrence with IFX, 38% with AZA, 70% with mesalamine	Clinical recurrence (CDAI > 150) at 6 months	Endoscopic improvement, changes in mucosal cytokine levels	6 months	No serious adverse events reported	IFX significantly reduced clinical and endoscopic recurrence and mucosal cytokine levels compared to AZA and mesalamine
Biancone et al., 2006 [38]	Endoscopic and clinical response	Pilot open-label study	Local injection of IFX	8	Endoscopic score improved in 3/8 patients, reduced number and extent of lesions in 7/8 patients	No clinical relapse observed during the follow-up period	Feasibility and safety of local iIFXnfliximab injection for CD recurrence	Clinical remission, histologic score, and assessment of local side effects	Median 20 months (range 14–21 months)	No local or systemic side effects reported	IFX injections were feasible and safe, with reduced lesion extent in most patients; further placebo-controlled studies needed to assess efficacy
Alves et al., 2004 [39]	Clinical recurrence	Retrospective cohort study	Immunosuppressive (IS) drugs (AZA, 6-mercaptopurine, or methotrexate) vs. Control (salicylates or no treatment)	26	N/A	Clinical recurrence rate at 3 years: IS group 25%, Control group 60%	Clinical recurrence rate at 3 years	Recurrence rate at follow-up, third intestinal resection rate	Mean follow-up of 80 ± 46 months	No specific IS complications reported	IS drugs lowered clinical recurrence and third resection rates after the second resection for ileocolonic anastomotic recurrence in CD patients
Dejaco et al., 2004 [40]	Endoscopic and clinical response	Open-label pilot study	(rhG-CSF)	5	Complete mucosal healing in 2 patients (40%); Partial response in 4 patients (80%)	All patients remained in clinical remission for 12 months	Complete mucosal healing (RSi0)	Intestinal permeability, cytokine levels, quality of life (IBDQ)	12 months	Transient headache, mild bone and muscle pain observed in 2 patients	rhG-CSF was well tolerated and demonstrated potential efficacy in treating severe endoscopic POR in CD patients
De Cruz et al., 2013 [41]	Endoscopic and clinical response	Multicenter randomized controlled trial	Immediate postoperative ADA vs. Step-up ADA at 6 months	60	43% endoscopic recurrence with immediate Adalimumab, 59% with step-up Adalimumab	32% complete mucosal normality with immediate ADA, 22% with step-up ADA	Endoscopic recurrence at 18 months	Severe disease recurrence rates, mucosal healing	18 months	Not specifically reported	No significant difference in recurrence; step-up anti-TNF therapy based on endoscopic findings viable for high-risk patients
Reinisch et al., 2008 [42]	Clinical and endoscopic response	Randomized double-blind double-dummy multicenter trial	AZA vs. Mesalamine	78	46.3% endoscopic improvement with AZA vs. 29.7% with mesalamine (ITT); 63.3% vs. 34.4% (completer analysis)	Not specified	Therapeutic failure (CDAI ≥ 200 or drug discontinuation due to lack of efficacy or intolerable adverse reaction)	Endoscopic improvement (≥ 1 point drop in RS)	52 weeks	Not specified	No significant difference in therapeutic failure rates; higher endoscopic improvement with AZA

Abbreviations: CD—Crohn’s disease, RS—Rutgeerts score, POR—postoperative recurrence, rhG-CSF—granulocyte colony-stimulating factor, AZA—azathioprine, IFX—infliximab, ADA—adalimumab, UST—Ustekinumab, VDZ—vedolizumab.

**Table 2:** Overview of the effectiveness of different drugs compared to IFX.

Treatment	Odds Ratio	95% Confidence Interval	Comparative Effectiveness to IFX	Comments
Infliximab (IFX)	Reference		Baseline	Widely used and well studied; serves as the reference treatment in this analysis
Adalimumab (ADA)	0.49	0.26–0.91	Less effective than IFX	ADA combined with thiopurine shows better outcomes compared to ADA alone
ADA + Thiopurine	0.25	0.08–0.83	Less effective than IFX	Combination therapy is more effective than monotherapy
Azathioprine (AZA)	0.16	0.03–0.80	Less effective than IFX	AZA shows lower effectiveness but may have a higher adverse event profile
Ustekinumab	9.3	1.06–81.50	More effective than IFX	Significant endoscopic and clinical success; favorable safety profile
Vedolizumab	17.35	2.03–148.66	More effective than IFX	High odds of achieving endoscopic response; promising alternative to anti-TNF therapies
Mesalamine	0.1	0.02–0.45	Less effective than IFX	Significantly less effective in inducing endoscopic response compared to IFX; safer profile but limited efficacy

## Data Availability

The data supporting this article are available within the manuscript.
